# Metamaterials and Metasurfaces: A Review from the Perspectives of Materials, Mechanisms and Advanced Metadevices

**DOI:** 10.3390/nano12061027

**Published:** 2022-03-21

**Authors:** Adnan Ali, Anirban Mitra, Brahim Aïssa

**Affiliations:** 1 Qatar Environment and Energy Research Institute (QEERI), Hamad Bin Khalifa University (HBKU), Qatar Foundation, Doha P.O. Box 34110, Qatar; adali@hbku.edu.qa; 2 Department of Physics, Indian Institute of Technology Roorkee, Roorkee 247667, India; anirban.mitra@ph.iitr.ac.in

**Keywords:** metamaterial, metasurface, metadevice, plasmonic, nanoparticle, photonic, crystal, lithography, electromagnetic wave, nanostructure

## Abstract

Throughout human history, the control of light, electricity and heat has evolved to become the cornerstone of various innovations and developments in electrical and electromagnetic technologies. Wireless communications, laser and computer technologies have all been achieved by altering the way light and other energy forms act naturally and how to manage them in a controlled manner. At the nanoscale, to control light and heat, matured nanostructure fabrication techniques have been developed in the last two decades, and a wide range of groundbreaking processes have been achieved. Photonic crystals, nanolithography, plasmonics phenomena and nanoparticle manipulation are the main areas where these techniques have been applied successfully and led to an emergent material sciences branch known as metamaterials. Metamaterials and functional material development strategies are focused on the structures of the matter itself, which has led to unconventional and unique electromagnetic properties through the manipulation of light—and in a more general picture the electromagnetic waves—in widespread manner. Metamaterial’s nanostructures have precise shape, geometry, size, direction and arrangement. Such configurations are impacting the electromagnetic light waves to generate novel properties that are difficult or even impossible to obtain with natural materials. This review discusses these metamaterials and metasurfaces from the perspectives of materials, mechanisms and advanced metadevices in depth, with the aim to serve as a solid reference for future works in this exciting and rapidly emerging topic.

## 1. Introduction to Metamaterials

Metamaterials are amongst the advanced materials made up initially with metal structures. However, there is a huge ongoing work on dielectric metasurfaces and metamaterials with the aim of replacing metal structures with dielectric ones in order to reduce the electromagnetic losses.

**Figure 1:**
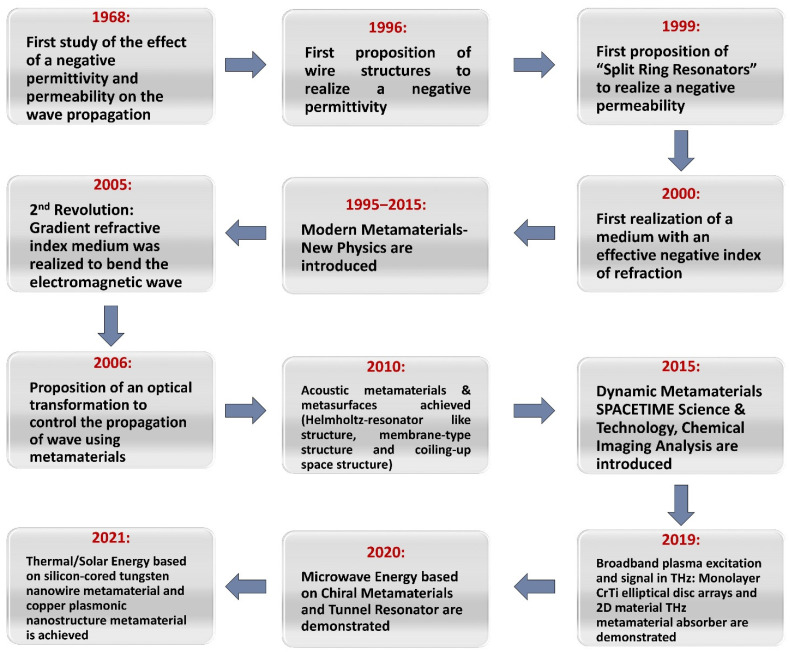
Schematic depicting the evolution of the field of metamaterials over the years, from 1968 to nowadays.

Metamaterials’ physical properties rely mostly on their structures. In 1968, Veselago [1] explored materials of negative permittivity and permeability. Such characteristics though are not present in naturally found materials and can only be generated in metamaterials. During the electromagnetic wave transmission such as wave propagation, the effects generated by metamaterials can be clearly observed. Evolution of the topic of metamaterials with time is presented in Figure 1. From an applications point of view, metamaterials could be used in devices such as antennas [2], photonic filters [3], integrated network sensors [4] or new superlayers for the microwave and terahertz fields [5]. The deep understanding of metamaterials offers fullness of novel options ranging from laboratories concepts to practical engineering applications.

The term “metamaterial” comes from the Greek words “meta” and “material”, while “meta” refers to something that is beyond usual, rearranged, changed or innovative. It is an engineered material intended to attain unique properties and capabilities which are absent in natural materials. The term metamaterial is introduced by Walser in 1999 [6]. Metamaterial cannot be obtained from any continuous and homogenous medium, that is why the metamaterials are always of a composite nature. Usually, metamaterials are constructed from discrete resonant micro- and nanometer-scale objects which mimic the electromagnetic reaction of atoms and molecules of natural substances to make them interact with light and other forms of energy in specific controllable ways.

## 2. Classification of Metamaterials According to Their Physical Properties

The core concept of metamaterial design is to craft materials by using artificially designed and fabricated structural units to achieve the desired properties and functionalities. These structural units—the constituent artificial ‘atoms’ and ‘molecules’ of the metamaterial—can be tailored in shape and size, the lattice constant and interatomic interaction can be artificially tuned, and ‘defects’ can be designed and placed at desired locations. By engineering the arrangement of these nanoscale unit cells into a desired architecture or geometry, one can tune the refractive index of the metamaterial to positive, near-zero or negative values. By taking into account the permittivity (ε) [7] and permeability (μ) [8] of a homogeneous material, the metamaterial classification was introduced by Veselago [1]. As a consequence, some abnormal physical phenomena occur if ε and μ are simultaneously negative, such as the reversal of the Snell Law [9], the reversal of the Cherenkov Effect [10], and the reversal of the Doppler Shift [11]. The relation between the refractive index (n), ε and μ component parameters are as follows:

(1) $$n=\pm \surd \mu_{r}\varepsilon_{r}$$

**Figure 2:**
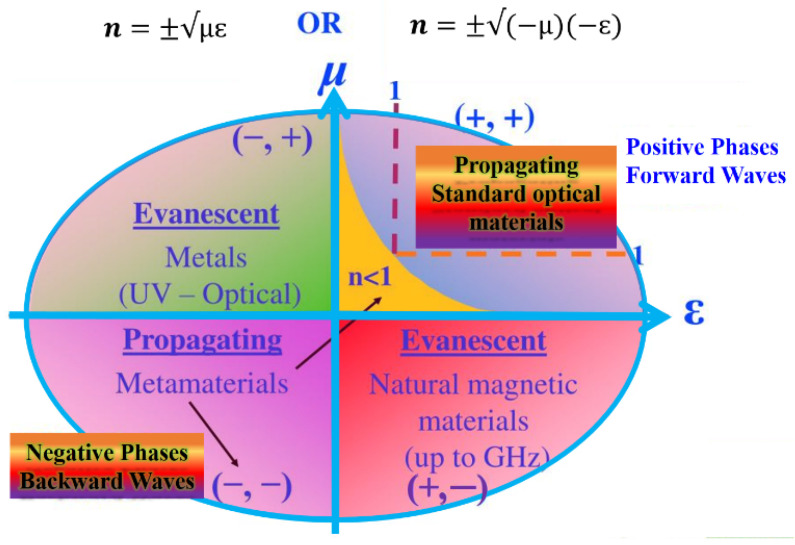
Summary of the metamaterial’s classification based on their permittivity (ε) and permeability (μ). Adapted from Ref. [16].

$\mu_{r}{\;\mathrm{and}\;\varepsilon }_{r}$ are the material relative permittivity and permeability, related to the free space permittivity and permeability by $\varepsilon_{o}=\frac{\varepsilon }{\varepsilon_{r}}$ = 8.854 × 10^−12^ F/m and μo =μμr = 4π × 10^−7^ H/m, respectively. From Equation (1), the value of refractive index “n” depends on the respective pairs of signs $\varepsilon_{r}$ and $\mu_{r}$. For example, flat lens with an isotropic materials ε_r_ = μ_r_ = −1 (refractive index n = −1). Therefore, the light transmission of the radiation from an object is unity for all Fourier components, even including evanescent waves that carry the information of high spatial frequency. In other words, flat lens can in principle form a perfect image of a point source. The classification based on the pair sign ε and μ is shown in Figure 2. Various metamaterials were developed with each quadrant corresponding to the structure.

Both parameters ε and μ are positive in quadrant I and are referred as double positive (DPS) or right-handed medium (RHM). Quadrant I materials are present in nature such as dielectric materials in which propagation of electromagnetic waves can take place. In quadrant II, where ε < 0 —negative, and μ > 0—positive, the corresponding materials are known as epsilon negative (ENG) medium, and are represented by an electric plasma, which support evanescent waves. Such medium has a frequency typically between 2 and 20 MHz. In many metals, the negative value of the electric permittivity occurs below the plasma frequency at the optical frequency range. For the frequencies lying below the medium frequency, the real part of electric permittivity is negative and dominates the imaginary part. The geometrical parameters of the structure determine the value of medium frequency [12]. The quadrant III, where ε < 0—negative, and μ < 0—negative, is known as double negative (DNG) or left-handed medium (LHM), and do not exist in nature. In quadrant IV, where ε > 0 —positive, and μ < 0—negative, the corresponding materials are termed as μ—negative (MNG), and represented by ferrite materials. In regions I and III, most waves can propagate, whereas in regions II and IV, evanescent waves can be found and are non-propagating [13,14,15,16,17].

The dimensionality of the range of components that form the bulk (3D) structures is another criterion for metamaterial classification. They are structures that have a large number of constituent elements in any given direction. The materials on the surface (i.e., 2D) are equivalent to the case where a thin film structure is made of 1–3 constituents only. Surface-type metamaterials are generally called metafilms or metasurfaces. Optical waveguide with nano inclusions and plasmonics and polaritonic nano-chains are considered as linear (1D) structures. As a matter of fact, optical waveguides are also identified as meta-waveguides [18,19,20].

Metamaterials in a functional applications field were extensively investigated, and included (but not limited to) light sources, sensors, memory, phase change, microelectromechanical systems (MEMS), nanoelectromechanical systems (NEMS), superconductors (based on negative index metamaterials), chiral metamaterials (intrinsic and extrinsic metasurfaces), dispersion metamaterials and artificial magnetism and transformation optical metamaterials [14,15,21,22].

The term metamaterial is also used in conjunction with artificially designed materials to exhibit novel properties of waves such as phononic metamaterials [23]. On the other hand, electromagnetic waves are stimulated by a different kind of non-electromagnetic waves such as the sound waves of piezoelectric and/or spin waves of nanostructured magnetic materials.

## 3. Evolution of Metamaterials

**Figure 3:**
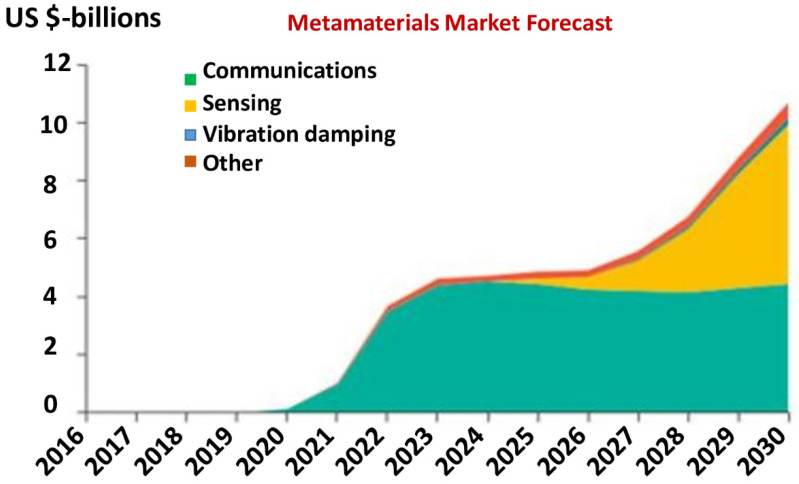
Metamaterials market forecast: metamaterial devices are poised to grow to $10.7 billion by 2030 in 5G networks, autonomous vehicles and connected vehicles. Adapted from Ref. [24].

In 2019, Vicari et al. [24] modeled the growth of metamaterial-containing devices across eight different applications (displayed in Figure 3). The model looked at potential addressable markets based on various parameters including the cost, maturity and performance. It is based on inputs from a wide range of primary and secondary research. Vicari and his team explicitly seized the markets for metamaterial components in communications, sensing and acoustic applications. Many other applications are likely to appear and were grouped together into an “other” category. Market forecast for metamaterials is put at $10.7 billion by 2030. Through 2025, communications uses are by far the leading growth driver; however, by 2030, sensing uses grow to be the largest segment, reaching $5.5 billion compared to $4.4 billion in communications.

**Figure 4:**
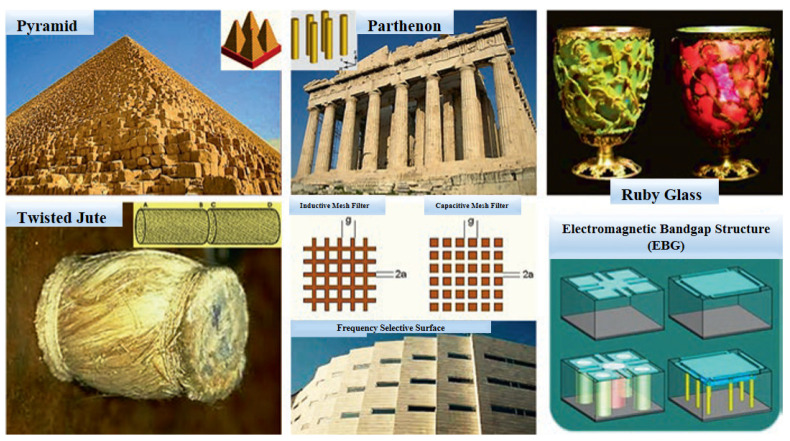
Examples of some “historical” original metamaterials, adapted from Ref. [26].

The origin of metamaterials could be linked to various examples of the pyramid brick wall, Parthenon columns and medieval ruby glass, as shown in Figure 4. Other “historical” examples of metamaterials are, for instance, the work on the rotation of the polarization plane through artificial twisted structures performed in 1898, and the artificial dielectric structures for microwave antenna lenses achieved in 1945. The modern (formally named) metamaterial was noticeable when Pendry et al. [25] anticipated that arrays of conducting wire can function with a negative value of effective permittivity at a relatively low frequency (<200 THz), whereas the split ring resonators (SRRs) can be utilized to facilitate a strong magnetic resonance which resulted in an effective permeability negative value. In 2000, by linking both structures and overlapping the negative frequency bands, a metamaterial carrying a negative refraction index was presented for the first time, where the directions of the wave vector and the energy flux, also known as backwards waves, were opposite in a negative index medium. These experiments have proven the predictions of Veselago’s 1968 negative index materials [1], such as negative refraction, Doppler’s reversed effect and Cherenkov’s reversed radiation [10].

### Metasurfaces

Depending on the materials employed and geometry, and per their frequency responses, metasurface-based device’s classification has been made as narrowband [3,27], broadband [28,29,30] and wideband [31]. Fano resonance is the typical case of narrowband application. The sharp resonance peak complemented by strong local field enhancement is amongst the most important aspects of Fano resonance [32,33]. It is the foundation of several practical applications such as nonlinear photonics and biological sensing. Metasurface dispersion for multiband and broadband applications can support super-resolution imaging beyond the diffraction limit, high-performance color filter, broadband absorbers and polarizers [31,34,35,36].

Metasurfaces are two-dimensional (2D) or planar versions of metamaterials dealing with subwavelength thickness [37,38,39]. Metasurfaces have unique abilities to block, absorb, concentrate, disperse or guide waves on the surface at a grazing incidence angle and in space at normal and oblique incidence, from microwave to visible frequencies. Surface waves can be well controlled by designing impedance cells to manipulate phase or group velocity [40,41]. These are patterned in such a way that may guide or separate waves in certain directions, and/or used to control scattering. By controlling the metasurface, unit cell sizes and shapes, multiple effective surface refractive indices can be achieved and different functions can be patterned on the surface. They can be used to design 2D microwave/optical lenses for antenna systems and planar microwave sources, such as Luneburg and fish-eye lenses [42].

**Figure 5:**
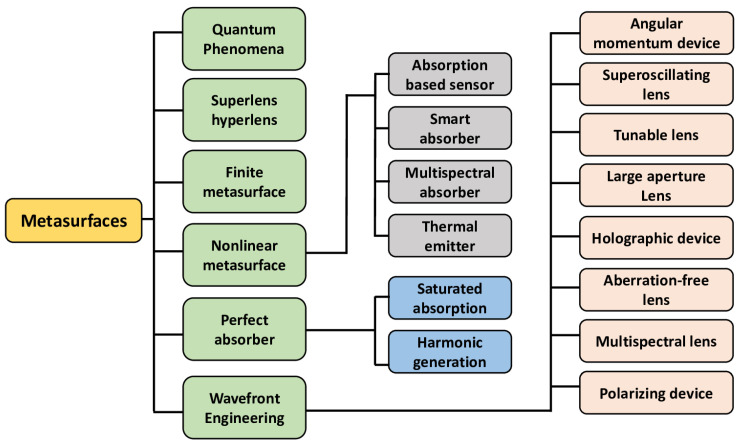
Chart summarizing the metasurfaces’ and metadevices’ applications.

The perspective of current implementation and growing metasurface and metadevice developments are shown in Figure 5. Different metasurfaces demonstrate different capabilities. The surface impedance can be modified and controlled by the structure of the cells that are widely applied to surface wave absorbers and surface waveguides. These metasurfaces also enable transmission and reflection to shape the beam.

Three-dimensional (3D) metamaterials, made often with periodic artificial materials composed of metals and/or dielectrics, have been widely studied owing to their unique interaction with electromagnetic waves that exceed the capabilities of naturally occurring or homogeneous materials [27,43,44]. Their exceptional ability to manipulate waves is due to their strong interaction with electrical and/or magnetic fields, typically provided by unit cell geometry-controlled resonant effects. These capabilities result in a broad range of applications such as antenna efficiency enhancement [45,46], ideal absorbers [47,48,49], superlenses [25,50], cloaking [51,52,53], elimination of scattering [28,54] and energy harvesting [16,55,56], among other applications in microwave and optical frequencies. When active and nonlinear components such as transistors [57,58], diodes [58,59], and varactors [60,61,62] are added to metasurfaces, novel tunability and switching abilities become possible.

## 4. Metamaterial Fabrication Techniques

Transmission line, resonant and hybrid methods are among the most common design approaches to efficiently fabricate metamaterials from a micrometer to nanometer scale. The implementation of hard materials such as dielectrics/metals into flexible materials, combined with the cutting-edge nanometer feature size techniques, has made it possible to fabricate stretchable devices across multidisciplinary.

### 4.1. Photolithography

**Figure 6:**
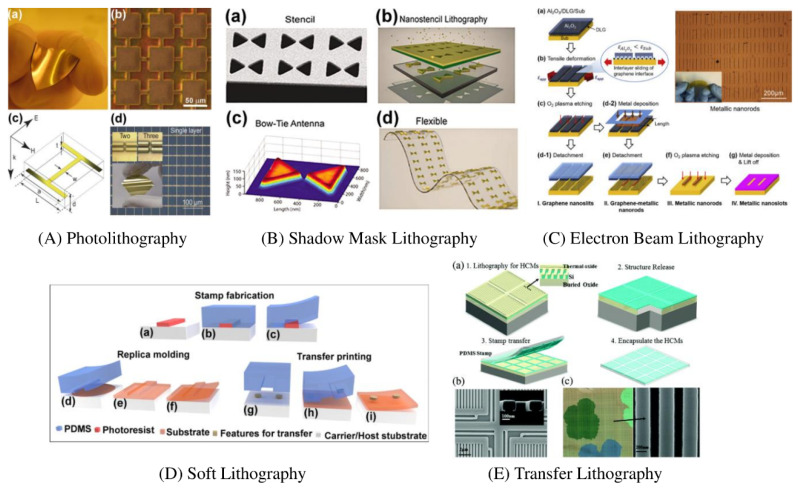
(**A**). Photolithography: Microfabricated terahertz metamaterials on flexible substrates, (**B**) Shadow Mask Lithography (SML): SML manufacturing sequence, (**A**,**B**) Reprinted with the permission of Copyright © 2018, Springer International Publishing AG). (**C**) Electron Beam Lithography (EBL): (**a**–**g**) Steps for the transferred double-layer graphene on a flexible substrate. (Reprinted with permission from Elsevier and Copyright Clearance Center), (**D**) Soft Lithography, (**a**–**c**) Fabrication of elastomeric stamps, (**d**–**f**) Replica molding (**g**–**i**) transfer printing, (**E**) Transfer Lithography: Flexible photonic metastructures (**a**) schematic for a high-contrast metastructure (HCM) preparation. (**b**) SEM images after etching. (**c**) Microscopic (**left**) and SEM (**right**) for the transferred HCMs on PDMS. (Reprinted with permission from Royal Society of Chemistry).

Photolithography is a technique of microfabrication, which can be used to synthesize metamaterials that work at terahertz frequencies. This technique has been commonly used in the manufacturing of single and multilayer (3D) metamaterials [63], since it is able to fabricate high-resolution subwavelength structures at wavelength 30 μm–3 mm with minimal complication [64,65]. Figure 6 provides an example of the reported metamaterial made by microfabrication onto flexible substrates. Resonators have been fabricated in the metal film deposited on spin-coated polyimide substrates. In order to prevent the delamination of metal structures, the micron resolution patterns produced by this technique are typically further coated with a thin film of polyimide. In general, microfabrication photolithography techniques are only appropriate for substrates that are organic and corrosive solvent resistant.

### 4.2. Shadow Mask Lithography

The shadow mask lithography (SML) is a technique which is acid-free manufacturing, produces micro- and nanoscale patterns in planar and multiple layers, similar to screen printing of conductive ink. This technique involves direct deposition of thin metal films, oxides or other dielectric particles on substrate using a pattern without any etching and photolithography [66,67,68]. The manufacturing sequence of the SML technology is shown in Figure 6A [69,70]. A stencil is fabricated usually by etching the silicon wafer or aluminum foil. The stencil is either directly in contact with the substrate or close to it. In order to exploit its inherent line-of-sight depositing characteristics, required metal or dielectric layers, primarily by evaporation of the electron beam, are then deposited. Features of up to 100 nm can be fabricated while using the SML on arbitrary substrates which are chemically sensitive or mechanically fragile (including plastic and polymeric substrates). This method permits large area fabrication of nano-patterning. The stencils are reusable, with high-performance pattern replication showing repeatable results. However, over repeated cycles of deposition the resolution of the recognized patterns often deteriorate due to stencil contact and proximity to the substrate during deposition [71].

### 4.3. Soft Lithography

Soft lithography (SL) is a class of technologies employed to fabricate or reproduce structures utilizing elastomeric stamps, molds and conformable photomasks. Because it uses elastomeric materials, most notably polydimethylsiloxane (PDMS), it is called soft. SL enables the multi-scale patterning on polymers or other substrates of micro/nanoscale patterns [72]. With high-resolution replica molding, any polymer compliant to the PDMS stamping, including non-photolithographic definable polymers, can also be patterned with high resolutions. A free-standing design is developed by fabricating an inverted copy of the stamp that can be detached from the carrier [70].

The transfer printing is another widely employed manufacturing technique. Characteristics of any required materials such as metals, semiconductors or functional oxides can be developed on a silicon substrate. It facilitates the application of well-known patterning technologies. The elastomeric stamp is used to “pick up” the patterns and then transfer to the substrate. It is useful and simple method but demanding highly monitored precision to transfer patterns to the target substrate with high adhesion [73]. Figure 6B presents a 3D modular transfer printing for assembly on universal substrates of heterogeneously integrated metamaterials/metadevices [73,74].

By using soft lithography techniques, plastic substrates’ limitations such as expansion at high temperatures, poor adhesion, low processing temperatures and chemical instabilities are eliminated. These techniques are also applicable to a wide range of structure sizes and allow non-planar surface patterning.

### 4.4. Electron Beam Lithography

Electron beam lithography (EBL) utilizes a focused electron beam wavelength at high accelerated voltages to achieve nanoscale structures (Figure 6C) [75,76]. Similar to traditional photolithographs where ultraviolet (UV) light is applied to a photo-resistant substrate, the EBL technique includes exposure by the high-energy electron beam to an electron beam resist, such as polymethyl methacrylate (PMMA). This leads to a division in the organic structure, where the area exposed to the e-beam fully dissolves in the developer solution. Then, metal/or dielectric layers are deposited, and the resist is dissolved to obtain the nanoscale patterns. The “lift-off” process is used in this technique, which means that the reverse design defined by the EBL must be the required pattern. In order to create nanoscale features under the diffraction boundary of the traditional photolithographic processes, EBL is highly suitable and does not require a physical mask for pattern transfers. The EBL technology is used for the fabrication of subwavelength resonators. Although EBL is a powerful technique for achieving nanometer and submicron characteristics, the development of high-performance large-scale metamaterials must overcome three major limits, namely the lengthy writing times because technique is serial; periodical stitch errors; and the lack of stability and potential astigmatism of the electron beam. Furthermore, serial patterning causes drifting beam instability, and a wide area of many steps is leading to a poor resolution and stitching defects. Secondly, stitching errors also adversely affect the reproduction ability of the patterns without a misalignment on a large area. Finally, the reliability, precision and stigma of electron beam whitening have a decisive effect on the technique’s efficacy. The blanket beam is an outer voltage source, which is used for switching on and off electron beams when moving nanoscale functionality. During the extensive writing times usually associated with this process, any variation will lead to incoherent exposure and introduces geometric errors [74,77].

### 4.5. Three-Dimensional Metamaterial Fabrication Techniques

The formation of multiple 3D metamaterials was primarily based on planar processing techniques. For instance, cloaking [51,52], imaging below diffraction limit [78,79], quantum levitation and sensing [80] can be successfully achieved by 3D terahertz or optical metamaterials. However, anisotropic responses often affect such multilayered metamaterials. This cannot be achieved by planar processes to generate metamaterials with epsilon (ɛ) and mu (μ) isotropic negatives. Additional innovations in manufacturing technology have been pursued such as specific additive production processes, to obtain isotropic response metamaterials for the subwavelength dimension. Such techniques include imprint lithography, vertical pillar superlattice, multiphoton polymerisation, electroplating in multilayer, inkjet printing and interference lithography [74,76,77,81,82,83,84]. 

**Figure 7:**
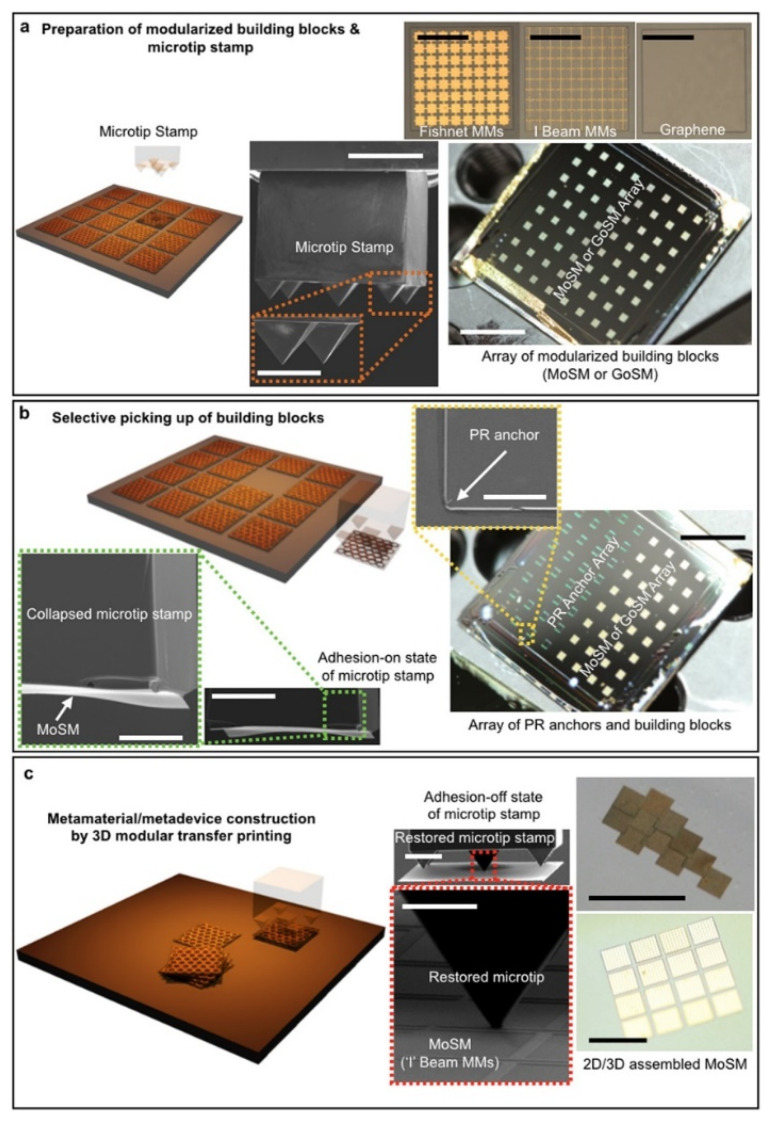
Heterogeneously integrated metamaterial/metadevice assembly on universal substrates by 3D modular transfer printing: (**a**) modularized basic building blocks preparation, (**b**) metamaterials on a silicon membrane (MoSM) and graphene on a silicon (GoSM) picking up by the 5-microtip stamp. (**c**) Three-dimensional modular transfer printing used to fabricate a metamaterial/metadevice. Two-dimensional and three-dimensional metamaterials and metadevices by assembling each individual building lock (i.e., MoSM or GoSM). (Reprinted with the permission of Copyright © 2018, Springer International Publishing AG).

Many other manufacturing techniques are developed in order to create a 3D split-ring resonator (SRR) metamaterial, by a combination of laser writing and chemical vapor deposition techniques [75,85]. Figure 7 shows a heterogeneously integrated metamaterial/metadevice assembly on universal substrates by 3D modular transfer printing. The technique of chemical vapor deposition grows a uniform metal film coating of the structure that cannot be obtained using other techniques. A focused ion beam (FIB) milling is another 3D manufacturing technique used for the fabrication of features with high aspect ratios at the nanometer scale [66,86]. The multiple-layer deposition of metal and dielectric layers was developed to create a 3D fishnet resonator. Etching of high aspect ratio nanometer-sized features was carried out by FIB milling. Furthermore, plasma etching can be used instead of the FIB milling. To produce sophisticated negative index metamaterials, a combination of printing techniques with other large-area lithography techniques can be used. Fabrication technique using 3D direct laser writing (DLW) may be utilized to fabricate metamaterial structures with complex geometry [87,88]. The DLW technique involves incident of a firmly focused laser beam on a diffraction-limited spot inside the photoresist’s volume. This enables the development of three-dimensional nanometer-scale features that cannot be manufactured using traditional lithography techniques. Whereas direct writing methods can reach optimized resolutions, they suffer from low output and are only suitable with a limited number of substrates. Multiple laser wavelengths technique can further improve the spatial resolution.

Another sophisticated fabrication technique of producing micrometer-scale 3D metamaterials is membrane projection lithography (MPL). Typically, this technique employs a patterned membrane layer built over a cavity to serve as a mask layer to the cavity’s underlying wall surfaces and bottom surface. A beam may be guided through the template opening onto the underlying surface, with the opening serving as a mask for regulating the area of the underlying surfaces on which any implantation occurs. MPL can revolutionize future 3D infrared and optical metamaterial structures with complex geometries, thanks to its high degree of control over the design and deposition of thin films [89].

Caputo et al. proposed a completely different approach to achieve hyper-resolution of a generic two-photon direct laser writing (TP-DLW) lithography process exploiting the extraordinary collimation of the writing laser light enabled by an optical epsilon-near-zero (ε_NZ_) nanocavity in a metal/insulator/metal/insulator (MIMI) configuration [90]. In particular, the proposed technique reaches its optimum when 2D and 3D complex nanostructures are under consideration. The proposed metamaterial is exploited to successfully fabricate 1D gratings with a height adjustable from 5 nm to 50 nm. Achieved results provided the driving force to implement a novel and highly effective approach for developing ultrathin all-dielectric metalenses. The ground-breaking advantages of this novel technique include cost effective and time effective single-step fabrication, nanometric sizes and all-dielectric composition. This technique can be used in applications across industry and scientific research, such as in miniaturized optical devices, advanced microscopy and flat-optics [87,88,90].

Though these techniques are promising in creating 3D metamaterial structures; however, they are not versatile and mature enough. Some of these presented methods are rather complex and need various manufacturing steps that impact their structural resolution, while others are restricted by the type of materials employed for the patterning process and also by the option of the appropriate substrates. Additionally, transfer techniques are based on the chemistry of the surface, and as a result, they are reliant on it. Overall, the use of complex techniques involving soft lithography and photolithography fusion to guide the pattern transfer to highly curved substrates remains a technological challenge to achieve high-resolution patterns of <0.1 μm.

## 5. Emerging Functional Metadevices

**Figure 8:**
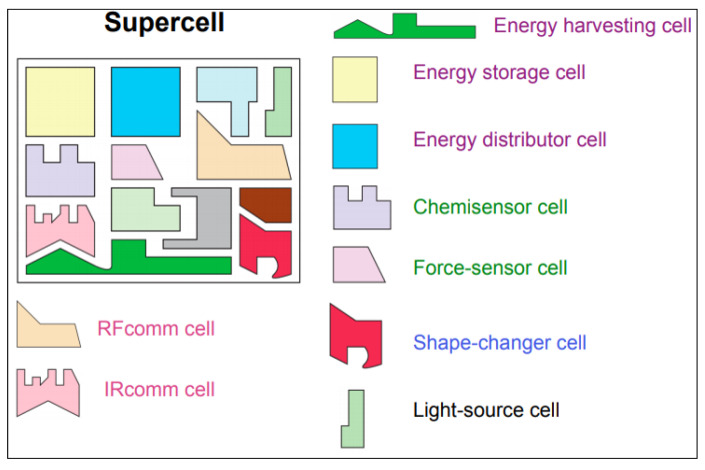
Schematic of the Nanoengineered Metamaterials and Super Cell.

Metamaterial research has shifted recently to the so called “integrated metadevices”, known as super cell [91] (Figure 8). Extensive studies have been conducted outside the electromagnetic photonic [52], terahertz [92] and microwave metamaterials utilizing phase-change devices [93], graphene [94], carbon nanoubes [95], semiconductors and liquid crystals [96]. The ultimate goal is to successfully obtain tuning, switching, nonlinear and sensing properties that are conducted by arranging the functional matter on the subwavelength level. Moreover, metadevices such as mechanical metadisplay, microelectricity, acoustics and metadisplays are also emerging and involving nonlinear and quantum superconductors, electrostatic and optomechanical forces and nonlinear metadevices with nonlinear lumped components [97,98,99,100].

### 5.1. Tunable and Reconfigurable Metadevices

**Figure 9:**
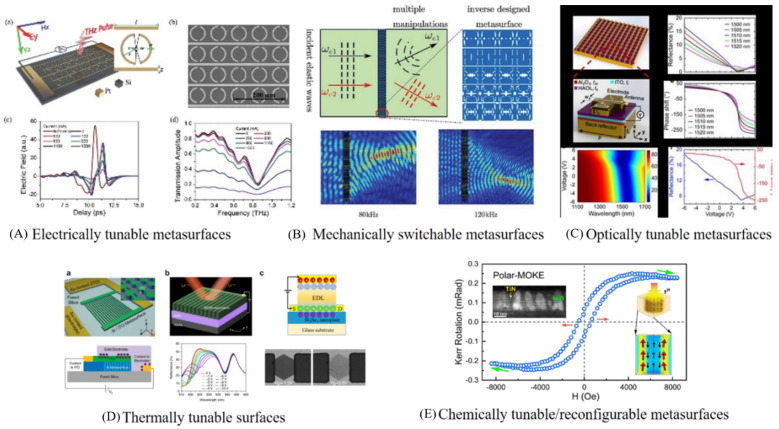
Reconfigurable and Tunable Metadevices (**A**,**B**) Reprinted with permission from John Wiley and Sons in Copyrights Clearance center, (**C**) Reprinted with permission from Copyright © 2020, American Chemical Society, (**D**) Reprinted with permission from Creative Commons Attribution 4.0 International License, (**E**) Reprinted with permission from Copyright © 2020, American Chemical Society.

Active metamaterial that is tuning its electromagnetic properties can be achieved by processing the size, shape and the composition, and by modifying the near-field interaction between individual meta-atoms or metamolecule resonators [101]. Reconfigurable and tunable metadevices can be classified as (i) electrically tunable metasurfaces (ETMs) [4], (ii) mechanically switchable metasurfaces (MSMs) [102], (iii) optically tunable metasurfaces (OTMs) [103], (iv) thermally tunable surfaces (TTMs) [97,103] and finally, (v) chemically tunable/reconfigurable metasurfaces [104,105], as presented in Figure 9.

Initially, by using transmission lines tuning, MEMSs have been utilized for electromagnetic metamaterials. Similarly, infrared and terahertz detectors for thermally activated structures can be applied. The strength of the dipole–dipole coupling of these MEMEs can be continuously adjusted by fine-tuning the distance between the two rings using the MEMS’ actuators, thereby enabling efficient electromagnetic adaptation reaction. The reconfiguration of the metamolecules may enable the polarization of anisotropic metamaterial to be switched independently [106,107,108,109].

There is consequently a significant impact on metadevices based on MEMS, NEMS, and micro-/nanofluidic technologies, where the mechanical nanoscale oscillation frequencies could lie within the GHz range, and the metamolecules can be operated at high bandwidth on the basis of subwavelength cantilevers.

### 5.2. Electro-Optical Metadevices

The main advantage of the electro-optical metadevices is their capability of reaching subwavelength deep modulation. They can operate at lower voltages offering an economical benefit compared with conventional technologies that use massive and costly electro-optical crystals. Initially, an active metadevice for the terahertz part of the spectrum was developed for a real-time radiation control using electric signals [110]. On a semiconducting substrate, Au metamaterial-arrayed metadevices were successfully fabricated to a manufactured electro-optical metadevice. An effective Schottky diode was formed by combining Au array and the substrate, in which the semiconducting nature can be controlled by injected and depleted carriers. The high-frequency electrical conductivity of the metamaterial is affected by an electrical signal and impacts its resonant response [111,112,113]. This approach offers the possibility to modulate the terahertz transmission by about 50%. The frequency and modulation bandwidths of this kind of metamaterial device are up to 10 MHz [92,112,114].

**Figure 10:**
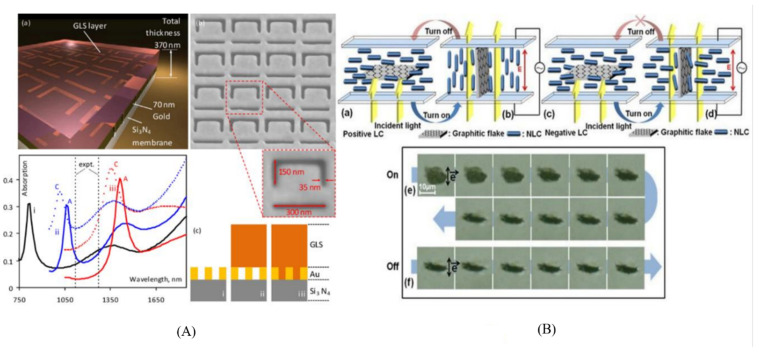
(**A**). Metamaterial electro-optic switch: (**a**) A hybrid device with planar plasmonics Au metamaterial coated with GLS chalcogenide glass on a SiN membrane. (**b**) Before GLS deposition, SEM of the asymmetric split-ring ASR slit array. (**c**) ASR structure computed optical absorption spectra without GLS film. Inside the figure (**c**), the black plot labeled **i:** with GLS flat film, the blue plot labeled **ii:** when the GLS also fills the slits in the gold; the red plot labeled **iii:** solid and dashed lines refer to the GLS phases of amorphous A and crystalline C, respectively. (Reprinted with permission with Rights managed by AIP Publishing) (**B**). Electro-optic behavior of the graphic flake display: in positive NLC (**a**,**b**) and (**c**,**d**) in negative NLC, before and after applying an electric field. (**e**,**f**) Showing the reorientation of a graphitic flake in the negative dielectric anisotropy LC as a result of the applied electric field (Reprinted with permission from OSA Publishing).

Graphene was also extensively used to build electro-optical metamaterials (Figure 10), and successfully applied as a modulator by modifying the electromagnetic response particularly in the infrared and terahertz domains [115,116,117]. Significant oscillator amplitude of the graphene plasmon resonances lead to noticeable optical absorption peaks at the room temperature. Moreover, the response of the graphene can be tuned efficiently by electrostatic doping over a broad frequency in the terahertz range.

### 5.3. Phase-Change Metadevices

For decades, semiconductor chalcogenide functionality has been used on optical disks supported by a change from amorphous to crystalline phases of a rewritable memory function. The features of polymorphic metals also provide a method of obtaining nanoscale, fast and energy-saving optical and plasmonic switching devices during this phase change. The changes are dependent on the stimulation/containment regime of the medium, and may be either reversible or irreversible [96]. Within an Al network, nonlinear optical and nonlinear plasmon nanocomposite material were developed through the introduction of grain-boundary gallium (Ga) [118,119]. In this context, two different phases of gallium intermediate coexistence triggered constant and reversible changes. This optical and temperature-sensitive composite metamaterial, formulates a mirror-like interface to silica and exhibits an extremely broadband transitional response to an optical excitement [2,120,121].

**Figure 11:**
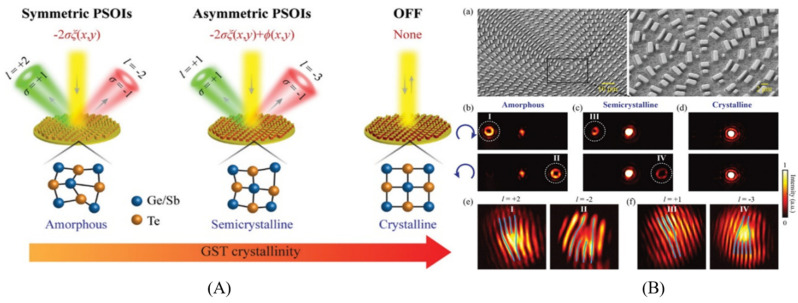
(**A**). The active SAM-to-OAM schematic conversion schematic activated with the suggested metasurface phase change. The crystallization level of GST can be changed among these three modes. (**B**). (**a**) The fabricated converter’s SEM images. (**b**–**d**) Diffraction patterns under RCP (**top**) and LCP (**bottom**) illuminating at various crystallization stages. (**e**,**f**) Interference patterns generated by interference with the OAM beams shown in (**b**,**c**) and the titled Gaussian beam, which is circularly polarized (Reprinted with permission from John Wiley and Sons and Copyright Clearance Center).

Figure 11 shows a conceptual illustration of multistate switchable photonic spin–orbit interactions that enable the conversion of various types of spin angular momentum (SAM) and orbital angular momentum (OAM) by controlling the level of crystallization of the Ge_2_Sb_2_Te_5_ alloy (GST) [122]. In symmetric and asymmetric modes, pseudo and real data can be coded, in conjunction with the degree of spin freedom. The metasurfaces are interpreted in the amorphous state by a simple geometric phase (Pancharatnam–Berry phase) [123], which leads to symmetrical SAM-to-OAM convert. A suitable stimulus is needed to interpret the accurate information to excite the GST phase transition from the amorphous to semi-crystalline state. Therefore, along with the geometric phase, a propagation phase is also introduced. The coupling of the two distinct phases enables the two opposite spin states to be regulated independently [124,125,126]. Two spin states at a given level of crystallization can be generated in the asymmetric mode. Several additional states of photonic spin–orbit interactions (PSOIs) can be recognized by optimizing the crystallization levels [127,128,129]. Furthermore, the PSOIs which is multistate switchable, have more information encryption freedoms and higher security. In addition, while several studies have also confirmed GST’s tremendous possibilities in active 1D wave-front modulation, such approaches are deduced mostly from discontinuity of the wave-front propagation phase and have no link with SAM and OAM [91,93,130].

### 5.4. Ultrafast Photonic Metadevices

In such devices, metallic nanostructured metamaterials combined with nonlinear and tunable dielectric or semiconductor films and handled by optically ultrafast pulses, offer extremely faster switching as compared to NEMS/MEMS. Intense light impacting the plasmon spectrum of a nanostructure triggers variations in the refractive index or absorption in the layer next to a plasmonic metamaterial array. It may contribute to a significant improvement in the hybrid’s resonant transmission and reflection. The main candidates are semiconductors and semiconductor multi-quantum well structures, graphene and carbon nanotubes [131] embedded in the metamaterial’s fabric [132].

The interaction between ultrafast optical pulses and metamaterials was first studied in order to see whether they could modulate their terahertz responses optically: shunting the metal split-ring network capacitive region by inserting optical carriers into the supporting ErAs/GaAs superlattice which recovery time at the ps scale causes a profound modulation of the terahertz transmission properties of the planar metamaterial [133,134]. Advancement in the field of plasmonic resonance due to metamaterial network can be employed in the optical part of the spectrum to increase the nonlinear response of the nearby layer of the semiconductor or dielectric. Fano-type resonances are promoted by a plasmonic nanostructure, where more than a ten times increase of the nonlinearity has been achieved. This technique permits the engineering of the nonlinearity of graphene at a predefined wavelength within a wide range of wavelengths, allowing for optical switching and pulse shaping applications [135,136,137].

**Figure 12:**
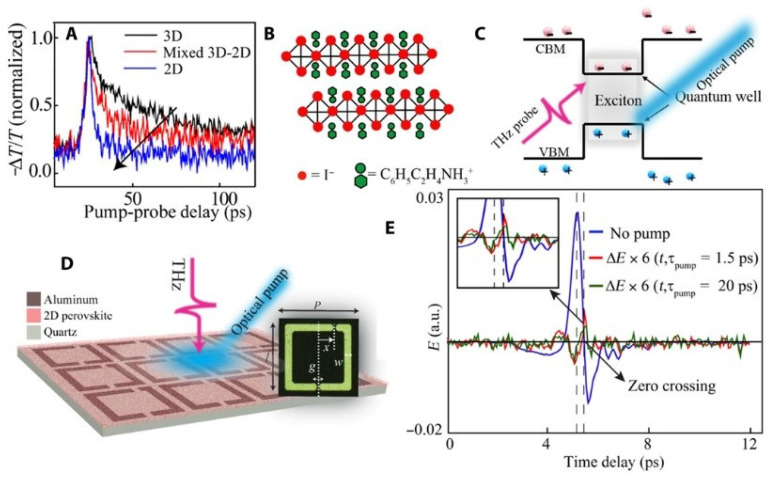
Two-dimensional perovskite ultrafast measurement of. (**A**) Three-dimensional perovskite (black solid curve), mixed 3D–2D perovskite (red solid curve), and pure 2D perovskite (blue solid curve) thin films free carrier excitation and relaxation dynamics. Using a 400 nm pump beam at 750 μJ/cm^2^, thin films are photoexcited. (**B**) Two-dimensional perovskite crystal structure’s schematic that makes QWs. (**C**) Scheme of the quantum confining of the pure 2D perovskite in the QW structure (**D**) The artistical example of the perovskite-coated hybrid metadevice with spin-coated 2D perovskite (thickness 60 nm) on top of TASR and photo-excited with a 400 THz optical pump beam. (**E**) THz electric field transmission (blue solid line) and THz photomodulation pulse ΔE(t) at τpump = 1.5 ps (red solid curve) and 20 ps (green solid line) measured in the 2D perovskite thin film (Reprinted with permission from Creative Commons Attribution-Non Commercial License 4.0 (CC BY-NC)).

Kumar et al. [5] recently reported a simple ultrafast THz photonic device where 2D perovskites and THz metamaterial were combined together for achieving enhanced THz waves modulation (Figure 12). Time-resolved THz spectroscopy (TRTS) measurement technique was used to investigate the free carriers and excitons dynamics in a pure thin film of 2D perovskite. In addition, by spin coating this 2D perovskite thin film onto THz metamaterials resonators, an ultrafast active metadevice for THz applications was fabricated.

### 5.5. Nonlinear Metadevices with Varactors

**Figure 13:**
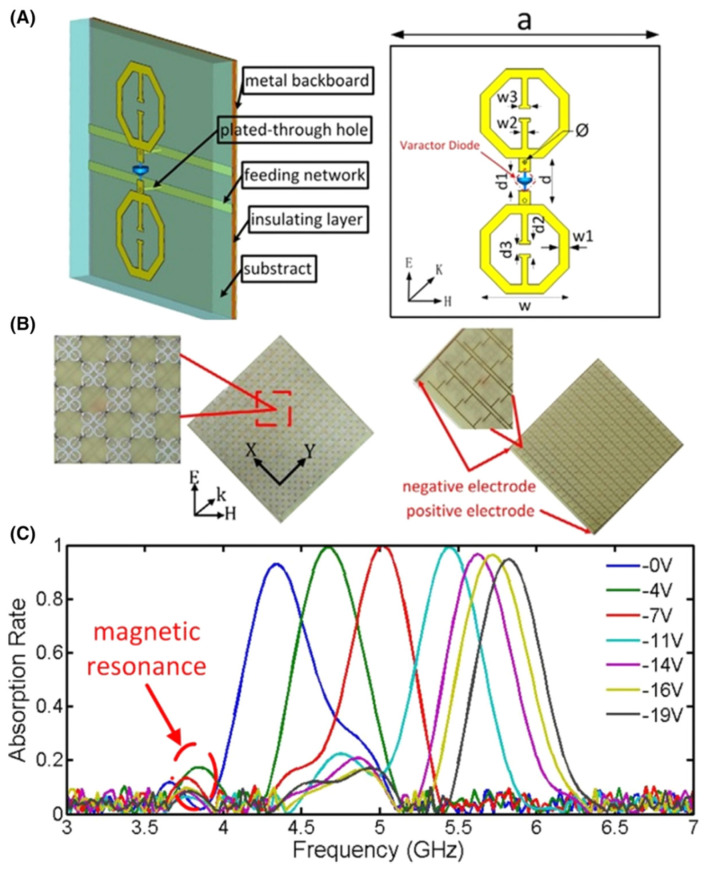
(**A**) Proposed metamaterial particle’s structure. (**B**) Fabricated sample front view and the varactor diodes feed network. (**C**) Absorption rates at different reverse bias voltages from 0 to 19 V. (Reprinted under the Creative Commons Attribution 3.0 license).

By exploiting the nonlinear behavior of lumped elements incorporated into LHMs, metamaterials can be tailored. Analogous approaches have been developed for other metamaterial systems. For instance, if positioned at a maximum point in the electrical field, split-ring metamolecule resonance can be modified by the addition of a series varactor diode with the resonator distributed capacitance. A split ring with embedded varactor presents second- and third-order nonlinearity at low powers, while the nonlinear response becomes multivalued or bistable at higher powers. In Figure 13, a nonlinear operating magnetic metamaterial is shown. For obtaining the tunability of metamaterials in a microwave domain, positive-intrinsic-negative (PIN) diodes or varactors are usually inserted into the passive particles compared to different tuning materials studied in terahertz range [113,138,139,140]. A tunable metamaterial absorber was suggested by Zhao et al. [60] using varactor diode and the proposed absorber tunability was confirmed by 1.5 GHz bandwidth measurements. Figure 13A shows the designed tunable metamaterial, which illustrates the structure of the particle wherein two similar electric-field-coupled-LC (ELC) spots are printed with same orientation on the substrate. Two ELCs are connected via varactor diode. As the structure resonant frequency is assessed using varactor diode lump parameters, under different reverse bias voltages the absorption frequency of the particle keeps on changing. On the substrate bottom, the feeding networks are printed and are used to communicate varactor diodes and feeding lines through holes. Figure 13C shows the results of the experiments as the bias voltage varies from 0 to 19 V, more than 90% absorption rate with the bandwidth 1.5 GHz from 4.35 to 5.85 GHz is achieved.

## 6. Photonic Band Gap

**Figure 14:**
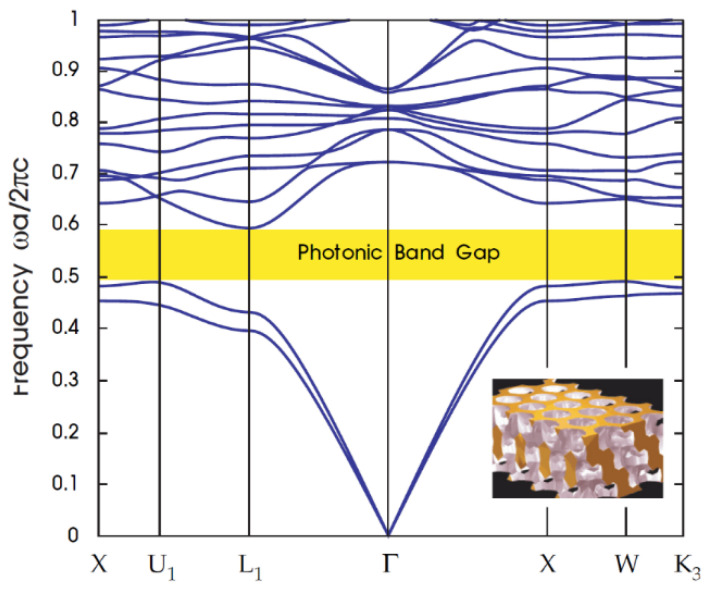
Representation of the photonic band gap sample (yellow colored area) on the photonic band diagram. The different directions along the crystal lattice indicated by the letters along the x-axis [144] (Reprinted with permission from Creative Commons Attribution-NonCommercial-ShareAlike 3.0 license.).

Photonic band gap (PBG) materials are artificial periodic structures in one, two, and three dimensions (1D, 2D, and 3D) with periods that are comparable with the wavelengths of electromagnetic waves (EMWs) [141,142]. The effect of a photonic band gap is quantified in terms of the local density of states, which describes the number of available electromagnetic modes in which photons may be emitted at the location of the emitter [143]. It contains a prohibited range of energy caused by variation in the refractive index, in which it can transmit light of a specific frequency and make it ideal for light harvesting applications, as shown in Figure 14. One can customize a structure’s specific band gap by replicating the periodicity of photonic crystals by establishing a pattern with repetitive domains, usually holes in a hexagonal or square structure that alternates between high and low dielectric constant materials.

**Figure 15:**
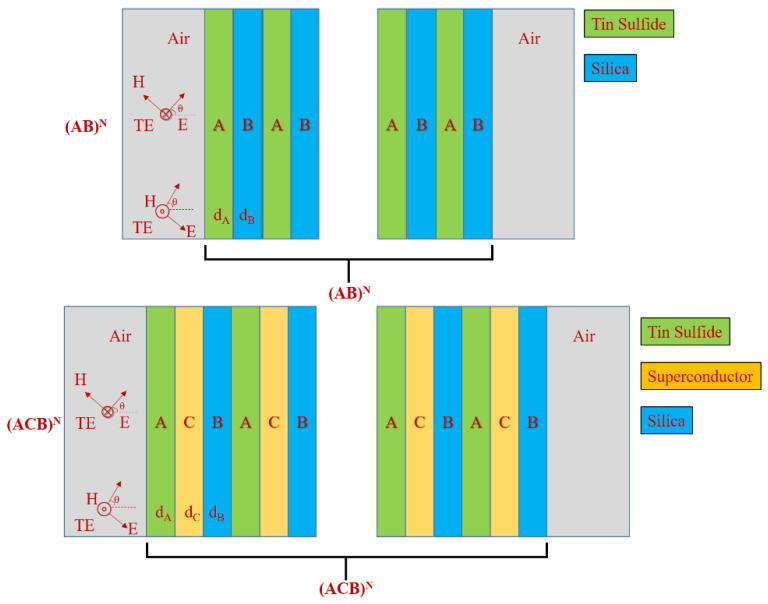
One-dimensional binary photonic crystal (1DBPCs) (AB)^N^ and one-dimensional ternary photonic crystal (ACB)^N^ representation, air is the background (Reproduced courtesy of The Electromagnetics Academy).

Photonic crystals (PCs) are materials that are periodically distributed in one, two or three spatial directions, showing stop bands or photonic band gaps (PBGs) [141,145]. The EMWs cannot propagate through the structure, with frequencies falling within PBG. In the PBGs, the localized states can be created by inserting defects into the periodic structures. PCs have been intensively investigated, due to their ability to control the propagation of light and for the possibility of many new optical devices. If the PBG can reflect an incident of EM waves at any angle with any polarization, an omnidirectional band gap (OBG) within a specific frequency range can be obtained with insignificant loss [142,143,144,145,146,147]. Fink et al. [143] pointed out in 1998 that one-dimensional PCs (1DPCs) may have OBGs, and the general conditions for obtaining OBGs are in 1DPCs. Since then, many scientists have drawn great interest from the OBGs in the 1DPCs [146,148,149,150,151,152,153]. Such an OBG is known to have potential applications [154,155], such as omnidirectional terahertz mirrors [156], controllable switching [157], tunable polarizer [158], narrowband filters [3] and optical refractometric sensing [159]. OBG width plays an important role in 1DPC omnidirectional reflector applications. However, the width of the OBGs is usually narrow in the one-dimensional binary photonic crystals (1DBPCs) formed by two different dielectric or metal-dielectric composites, making those structures inefficient as total reflector mirrors in application, as shown in Figure 15. Some methods have been proposed to extend the frequency range of OBGs, such as increasing the contrast of dielectric functions among PC composites [143,146], using a chirped PC [160] or photonic heterostructures [161,162,163], or introducing the disorder into periodic structures [164,165]. Additionally, being put forward in recent years is one-dimensional ternary photonic crystals (1DTPCs) to obtain the extended OBGs [166,167,168,169,170,171]. 1DTPCs comprise three layers of material over a lattice period. Awasthi et al. [168] demonstrated that when the structure was modified by sandwiching a thin layer of ZrO_2_ between each two layers, the wavelength range of OBGs can be increased by 108 nm. Wu et al. [170] have shown that the OBGs in the ternary metal-dielectric PC can be significantly enlarged. Xiang et al. [167] found that sandwiching the third material between the two single negative materials enlarge the zero-effective-phase bandgap. Kong et al. [171] introduced an OBG on 1D ternary plasma PC. In addition, some 1DTPC-based applications are the tunable optical filter [169] and optical sensing device [151].

### Photonic Band Gap Materials to Enhance Solar Cell Efficiency

**Figure 16:**
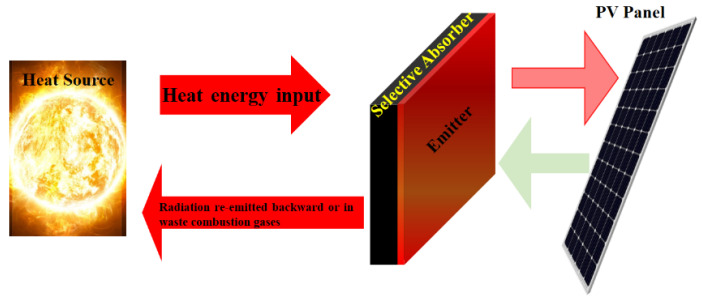
Illustration of a conversion system for TPV energy. The thermal radiation of the sun heats an intermediate absorber. Filter-transmitted radiation from the emitter illuminate the photovoltaic (PV) cell.

The most widely used power systems are photovoltaic solar energy conversion ones. However, these devices are suffering from relatively a low conversion efficiency. This is due to the wavelength mismatch between the narrow wavelength band associated with the energy gap in the semiconductor and the wide band of the sun’s (black body) emission curve. The loss of energy is linked to long wavelength photons with less energy stimulating the electron’s holes all over the energy gap (i.e., losing 20% in silicon) and photons having short wavelengths, stimulating pairs with energy over the gap (i.e., losing 32% in silicon). The efficiency of the thermophotovoltaic (TPV) system may be increased by using a spectrally dependent coupling between the absorber and the cell to recycle the photons with a frequency larger than the band gap frequency of the solar cell, as shown in Figure 16.

In this approach, materials of the specific PBG are designed in such a way to obtain highly efficient solar cells [141,145]. These are a new class of periodic materials which enable accurate control of all properties of EMWs [144,172]. A PBG, similar to the electronic band gap in semiconductor crystals, occurs in a periodic dielectric or metallic media. The electromagnetic radiation light cannot propagate within the PBG spectral range. Through the engineering of the photonic dispersion relationship, the ability to tailor the properties of the electromagnetic radiation in a prescribed way allows the design of systems that accurately control the emission and absorption of light. This gives rise to new phenomena including inhibition and enhancement of spontaneous emission [145], strong localization of light [141], formation of atom-photon bound states [173], quantum interference effects in spontaneous emission [174], single atom and collective atomic switching behavior through coherent resonant pumping and atomic reversal without fluctuations [175]. Feng et al. [176] reported the performance improvement of a near-field TVP device by a back surface reflector. As photonic crystals are the leading candidates for frequency- and angular-selective radiating elements in TPV devices. Therefore, employment of such photonic crystal-based angle- and frequency-selective absorbers facilitates a strong enhancement of the conversion efficiency of solar cell devices without using concentrators. The increment in efficiency is different for different materials, strategy (i.e., used as emitter, absorber or reflector) and temperature of the cell/system. As a matter of fact, using as back surface reflector (BSF), efficiency increased from 16.4% to 21% and power output has increased by 10%. Bierman et al. [177] reported for solar TPV, the 6.8% efficiency increase of the solar-to-electrical by pairing a one-dimensional SI/SiO_2_ photonic crystal selective thermal emitter and a tandem plasma-interference optical filter by spectral enhancement. Bhatt et al. [178] reported a STPV system with the overall conversion efficiency of 8.4% by using a nanostructure-based spectrally selective emitter/absorber. The spectral properties of the emitter were optimized to maximize the output power density from the TPV cells.

Changes in the spontaneous emission rate of atoms inside the photonic crystal structure in turn determine significant changes in thermal radiative processes. Thermal radiation is simply thermally driven by spontaneous emission and in thermal equilibrium with its surrounding material. In 1999, Cornelius and Dowling proposed the use of PBG materials to alter thermal emission [179]. Two alternative approaches were explored: a method based on a passive lossless PBG thin-film coating over the absorber, and an approach using an active PBG material made from an absorbent medium. In 2000, the thermal emission modification was experimentally demonstrated using a thin 3D photonic crystal slab on a silicon substratum [180]. Pralle et al. [181] used a PBG technique to prove a thermally excited, narrow-band, mid-infrared source. Recently, researchers at Sandia Labs demonstrated a high-efficiency TPV system using tungsten photonic crystals [182,183]. These studies suggest that it is possible to achieve dramatic modifications of Planck’s black body radiation spectrum by optimizing the coupling of the multi-mode radiation field of a PBG material and a spatially extended collection of atomic or electronic emitters [179,182]. The thermal emission of radiation is strongly suppressed in the PBG spectral range, whereas the thermal emission of radiation is resonantly increased up to the black-body limit for specific frequencies in the permitted photonic bands which correspond to the transmission resonances of the photonic crystal.

For important technological applications, such as low-threshold micro-lasers [18,184], ultra-fast all-optical switches and micro-transistors [185,186,187], these remarkable phenomena have attracted considerable interest.

## 7. Solar Cell Efficiency Enhancement and Negative Refractive Index Materials

In the weak absorption spectral domain, solar cells of planar thin-film configuration can benefit from resonance of Fabry–Perot to perform more effectively [130,188,189,190]. Similarly, spectral width significantly reduces for a thicker solar cell and their number increases, both being disadvantageous. There have been reports that a single Fabry–Perot resonance, which is spectrally broad, can be obtained from an optically alternate material, namely a material that has a negative but otherwise similar refractive index to the material of the solar cell with the same impedance. This is critical for improving the absorption across the interested spectral domain. Even though, the material assumed with idealistic properties cannot be obtained; therefore, nanostructures such as photonic crystals engineered for dispersion, can be manipulated by actual application. Their optical response may be comparable to the desired within the acceptable spectral range.

In solar cells the photon management contributes to improving spectrally and spatially the absorption of the incident sunlight [191,192]. This concept was under active investigation during the last decade. In this respect, numerous advanced micro- and nano-optical principles have been extended efficaciously to the photovoltaic domain. It usually goes further than conventional methodologies in which the incident of sunlight is only dispersed in macroscopic structures in order to maximize the efficacious path length [193,194]. Concepts used in these new methods are namely photonic crystals [195,196], metallic nanoparticles [197,198,199], waveguides [200,201] and diverse nanoshapes [202,203,204]. In the end, all these approaches focus on reducing PV material thickness without performance degradation. This minimizes recombination losses in volume, reduces material usage and accelerates the deposition time, whereas maintaining the level of absorption remains sufficiently high. Metamaterial’s field may allow a degree of freedom to work along two directions, such as properties for a specific structure are assessed and implemented for other purposes, e.g., to change the local state density [205] or to increase absorption [49,206]. On the other hand, a lot of essential work has been achieved in the opposite direction.

## 8. Metamaterials and Metasurfaces: Glimpses of New Trends

### 8.1. Terahertz Metamaterials

Terahertz (THz) radiation in the electromagnetic spectrum with its non-ionizing property has the ability to sense materials with an extremely high accuracy. THz devices have potential applications in diverse areas such as imaging, high-resolution spectroscopy and biomedical analytics. The idea to realize the optically reconfigurable metasurfaces based on phase-change material was proposed for multifunctional rewritable metadevices through laser writing that would open up a new avenue for real application of metamaterials. However, it is still not possible to realize the real-time control with the optical writing approach. Metasurfaces have provided a novel route to control the local phase of electromagnetic radiation through subwavelength scatterers where the properties of each element remain passive. A passive metasurface design can only achieve a specific functionality as it is extremely challenging to reconfigure each element that contributes toward the control of the radiation. To add more insights to this article, especially with the context of this planetary pandemic, some works in this particular topic of metamaterials should be highlighted. Indeed, in the diagnosis of severe contagious diseases there is an urgent need for protein sensors with large refractive index sensitivities. Current terahertz (THz) metamaterials cannot be used to develop such protein sensors due to their low refractive index sensitivities. Silalahi et al. [207] proposed an efficient method based on a patterned photoresist to float the split-ring resonators (SRRs) of a terahertz metamaterial at a height of 30 μm from its substrate that is deposited with complementary SRRs, and is compatible with all geometrical structures of terahertz metamaterials to increase their refractive index sensitivities. This floating terahertz metamaterial has demonstrated an extremely large refractive index sensitivity of 532 GHz/RIU and was tested successfully to sense bovine serum albumin (BSA) and the protein binding of BSA and anti-BSA as BSA, and anti-BSA solutions with low concentrations that are smaller than 0.150 μmol/L.

On the other hand, anapole metamaterials have recently attracted increasing attention owing to their unique nonradiating and nontrivial properties. Even though anapole modes have been demonstrated in metamaterials with three-dimensional structures, the design and realization of planar anapole metamaterials in a wide frequency range is still a big challenge. Li et al. [208] proposed and experimentally demonstrated a planar anapole metamaterial consisting of dumbbell-shaped apertures on a stainless-steel sheet at terahertz frequencies, and the planar metamaterial was even able to generate a resonant transparency in the terahertz spectrum due to the excitation of the anapole mode.

Zhang et al. [209] proposed a scheme based on a microelectromechanical system (MEMS) to reconfigure the resonance and radiation phase via control of each dipolar element. The suspension angle of the individual bimorph cantilever in air can be precisely controlled through electrostatic actuation that determines the operative phase diagram of the metadevice. The dynamic polarization conversion is demonstrated through global control. In addition, it is proposed that a multifunctional operation such as dynamic wavefront deflection and rewritable holographic display can be accomplished by using 1D and 2D control of the cantilever array when each cantilever in the MEMS metadevice array is uniformly and accurately controlled in the large-area samples [5]. Such a rewritable proposition can enable a myriad of applications of MEMS-based metadevices in polarization-division multiplexing and dynamic flat lenses. As an alternative, microelectromechanical system (MEMS) metamaterials have enabled the dynamic control of electromagnetic waves ranging from microwave to infrared through thermal bimorphs, comb drives, out-of-plane deformable cantilevers and electrostatically actuated beams [210].

### 8.2. Chiral Metamaterials

Chiral metamaterials have greatly impacted the field of optical sensing over the past decade [211,212]. To improve the sensitivity of chiral sensing platforms, enhancing the chiroptical response is necessary. Metasurfaces, have attracted significant attention because of their ability to enhance the chiroptical response by manipulating amplitude, phase and polarization of electromagnetic fields [22]. They can exhibit chiroptical phenomena, such as the difference in the propagation velocity, known as optical rotatory dispersion (ORD), and the difference of absorption, known as circular dichroism (CD), between left and right circularly polarized light [213,214,215]. 

The field of chiral plasmonics has met huge progress with machine-learning (ML)-mediated metamaterial prototyping. Ashalley and coworkers [216] presented an end-to-end functional bidirectional deep-learning (DL) model for three-dimensional chiral metamaterial design and optimization, exploring in detail the nontrivial relationship between the metamaterials’ geometry and their chiroptical response, eliminating the need for auxiliary networks or equivalent approaches to stabilize the physically relevant output. This model efficiently explored the sensing of biomolecular enantiomers and showed a promising potential to other applications including photodetectors, polarization-resolved imaging and circular dichroism (CD) spectroscopy.

### 8.3. Acoustic Metasurfaces

The acoustic metasurfaces field has emerged in recent years and is propelled by the desire to control the propagation of acoustic waves by compact structures [217]. The strategy for the design of acoustic metasurfaces depends on the targeted physical properties and applications (reflection, transmission and/or absorption). In the designs of acoustic metasurfaces for specific applications, precise control of the phase and amplitude of acoustic wave propagation is required. A thorough assessment of the design choice should take place before tackling any specific applications. The main metasurface designs are based on the Helmholtz resonator-like structure [218], membrane-type structure [219] and coiling-up space structure [220].

### 8.4. Bianisotropic Metasurfaces

Metasurfaces which exhibit magnetoelectric coupling are called bianisotropic metasurfaces [221]. Bianisotropic materials acquire magnetic (electric) polarization when excited by electric (magnetic) external field. Thus, the term “bianisotropy” implies double (“bi-”) polarization mechanism and anisotropic response. Optically thin composite layers can be modeled as electric and magnetic surface current sheets flowing in the layer volume in the metasurface plane. In the most general linear metasurface, the electric surface current can be induced by both incident electric and magnetic fields. Likewise, magnetic polarization and magnetic current can be induced also by external electric field. Bianisotropy is not always attributed to spatial dispersion. Alternatively, nonreciprocal bianisotropic effects can be achieved, for example, in composites containing both magnetically and electrically polarizable components which are coupled via their reactive fields and experience influence of some external time-odd bias field or force [222,223,224]. Nonreciprocal bianisotropic metasurfaces can be used to design, for instance, various types of isolators with simultaneous control of amplitude and polarization of transmitted waves [222,223,224].

### 8.5. Quantum Photonics Metasurfaces

An increasing interest in bringing novel functionalities enabled by flat photonics to the realm of quantum optics is recently noticed. Quantum optical technologies require sources of single photons, entangled photons and other types of nonclassical light, as well as novel methods of detection [88,185,225]. The quantum states could be based on different degrees of freedom of light polarization, direction and orbital angular momentum. Metasurfaces have a real potential for the realization of each of these states. Rapid progress in the development of metaphotonics allowed bulky optical assemblies to be replaced with metasurfaces, opening a broad range of novel and superior applications of flat optics to the generation, manipulation and detection of classical light [226,227,228,229,230].

### 8.6. Liquid Crystal-Based Metamaterials

Active liquid crystals (LCs)-based metamaterials have been attracting an increasing amount of attention in the past few years because of their controlled characteristics leading to emerging applications, including modulators, switches and filters. The use of LCs allows for the achievement of metadevices with dynamic functions induced by electro-, magneto-, photo- and temperature-sensitive properties. Kowerdziej et al. [231] reported on the shortening of switching times of various soft-matter-based tunable metamaterials to improve the functionality of modern active devices. As a matter of fact, the frequency-convertible dielectric anisotropy of the dual-frequency mixture has shown to provide the opportunity to create a fast-response in-plane switching metasurface at the nanoscale, which could be tuned by an electrical signal with different frequencies [232,233,234].

### 8.7. Non-Hermitian Photonics and Metamaterials/Metasurfaces

In quantum mechanics, hermiticity is usually assumed implying a closed system. Hermitian systems exhibit energy conservation, real eigenvalues and physically meaningful observables. If physical systems are allowed to exchange energy with their environments, these properties are no longer guaranteed. Such open or non-Hermitian systems generally have complex eigenvalues and may exhibit exceptional points [235,236,237]. Systems tuned to an exceptional point are extremely sensitive to perturbations [238,239]. In recent years, there has been tremendous progress in the theory and experimental implementations of non-Hermitian photonics, including all-lossy optical systems as well as parity-time symmetric systems [236,240]. Parity-time (PT) symmetry is a fascinating concept to make sense of non-Hermitian Hamiltonians. In particular, non-Hermitian concepts can be translated to electromagnetic structures by means of spatial modulation of loss and gain, which is becoming technologically viable in artificial materials and metamaterials [241]. Nowadays, materials and their geometry make up the tools for designing nanophotonic devices, but in the past to design the devices, focus used to remain usually on the real part of the refractive index of materials and the absorption, or imaginary index, was tolerated as an undesirable effect. However, a clever distribution of imaginary index of materials offers an additional degree of freedom for designing nanophotonic devices. Such configurations can be synthesized by establishing an even distribution in the real part of the refractive index while imposing an antisymmetric gain/loss profile, associated with the imaginary part of the refractive index [240]. The intrinsic capability of photonics of creating and superposing non-Hermitian eigenstates through optical gain and loss is ideal for exploring various non-Hermitian paradigms. PT metamaterials and metasurfaces may operate in microwave frequencies and higher, suppress losses and provide tunability through the natural appearance of a transition to a PT-broken phase. These systems enable new pathways for metasurface design using phase, symmetry and topology as powerful tools [242]. The research area of non-Hermitian photonics is relatively new and it is still largely unexplored. Novel theoretical ideas combined with new experimental schemes are expected to produce more surprising results, leading to altogether different, previously unknown, means for controlling light-matter interactions both in the classical and the quantum regimes.

### 8.8. Metamaterials-Based Coherent Perfect Absorber

A coherent perfect absorber is a system in which the complete absorption of electromagnetic radiation is achieved by controlling the interference of multiple incident waves. Interference is a ubiquitous wave phenomenon occurring whenever two or more coherent waves overlap, leading to a spatial redistribution of energy. Interference underlies the operation of numerous devices, in optics and photonics concerns the role that interference has in regulating light absorption. In particular, it is possible for a device to perfectly absorb an incident electromagnetic wave if all the wave components scattered by the device destructively interfere. Coherent perfect absorption (CPA) is a multichannel waveform shaping protocol that leads to the complete extinction of a monochromatic radiation when it enters a weakly lossy cavity [243,244]. Perfect absorption of light is of great importance in a variety of applications ranging from sensing to stealth technologies [245,246]. Achieving perfect absorption at subwavelength scale is particularly important for nanophotonic and electromagnetic applications. In recent years, various approaches of designing ultrathin perfect absorbers have been proposed, including thin films, metamaterials and metasurfaces [247]. However, it was found that the incident energy could be perfectly absorbed under incidence on opposite sides of an absorber. This interference-assisted absorption is known as “coherent perfect absorption”, and was experimentally demonstrated in a silicon slab under coherent monochromatic illumination [243,244,248].

## 9. Summary

The development of active metasurfaces and metamaterials to replace conventional optical elements is still in its early stages, although the field of metasurfaces is rather well established. In this review, we have reported the most recent and most relevant literature works related to the exciting field of metamaterials. Focus was put on metamaterials classification, evolution of metasurfaces, emerging metadevices and their mechanism of functioning. Traditional and new emerging fabrication techniques for metasurfaces are summarized with their associated advantages and limitations. Similarly, 3D metamaterial fabrication has been discussed. We have shed light onto photonic band gap tuning via the use of different metasurfaces to improve the absorption spectrum for specific applications such as enhancing solar cell efficiency. In photovoltaics, the level of light absorption can be commonly achieved by reducing the thickness of the absorption layer and minimizing the recombination losses. We have seen that these recommendations might be achieved through use of photonic crystals, metallic nanoparticles, waveguides and diverse nanoshapes. Thus, the door to metamaterials is fully opened now for the achievement of tangible innovative optoelectronic devices.
